# Clinical study on the efficacy of postural control combined with electroacupuncture in treating dysphagia after stroke

**DOI:** 10.3389/fneur.2024.1296758

**Published:** 2024-04-16

**Authors:** Yanli Wu, Zhongwen Zhang, Qing Li, Xiu Yuan, Jiange Ren, Yulin Chen, He Zhu

**Affiliations:** ^1^Central People’s Hospital of Zhanjiang, Zhanjiang, China; ^2^Gezhouba Central Hospital of Sinopharm, Yichang, China; ^3^Macheng Hospital of Traditional Chinese Medicine, Macheng, China; ^4^Liyuan Hospital, Tongji Medical College, Huazhong University of Science and Technology, Wuhan, China; ^5^Caidian District People’s Hospital of Wuhan, Wuhan, China

**Keywords:** stroke, rehabilitation, dysphagia, postural control, electroacupuncture

## Abstract

**Objective:**

To evaluate the clinical effectiveness of combining postural control with electroacupuncture in the treatment of dysphagia following stroke, with the goal of establishing a solid clinical foundation for this therapeutic approach and investigating potential mechanisms to stimulate additional research and progress in post-stroke dysphagia management.

**Methods:**

138 patients who met the diagnostic and inclusion criteria were enrolled and divided into control group and observation group. Both groups received conventional rehabilitation training. Additionally, the control group received swallowing training and diet optimize, while the observation group received swallowing training, diet optimize, posture control as well as electroacupuncture therapy. After four weeks, swallowing function was assessed and compared between the two groups using the Standardized Swallowing Assessment (SSA) score and water swallowing test (WST).

**Results:**

Patients who underwent postural control therapy in combination with electroacupuncture demonstrated significantly higher treatment efficacy compared to the control group. Notably, The SSA score and WST score in both groups decreased significantly, and the observation group showed more improvements in aspiration compared to the control group.

**Conclusion:**

The integration of posture control, electroacupuncture, and conventional rehabilitation training can effectively lower the degree of post-stroke swallowing disorders, restore swallowing function, and significantly reduce the occurrence of complications such as aspiration, fever, and nutritional disorders. Moreover, this approach significantly improves the quality of life of patients and is more effective than conventional rehabilitation training in treating post-stroke swallowing disorders.

**Clinical trial registration:**

https://www.chictr.org.cn/, Identifier ChiCTR2300075870.

## Introduction

Stroke frequently results in a range of functional impairments encompassing movement, sensation, swallowing, speech and language, cognition, mood, excretion, and cardiopulmonary functions (1–3). Post-stroke dysphagia (PSD) represents a prevalent complication, affecting an estimated 28% to 67% of stroke patients (4). Moreover, individuals with dysphagia following stroke often endure persistent swallowing difficulties, persisting up to six months post-onset (5). Neuromuscular Electrical stimulation (NMES), Pharyngeal electrical stimulation (PES) and modified Pharyngeal electrical stimulation (mPES), also have been reported as an effective treatment (6, 7). Nevertheless, the exact mechanism of NMES, PES and mPES is unclear, and there has been little consensus on optimal electrode placement as well as proper frequency and intensity for stimulation (8).

Acupuncture, rooted in the meridian theory, entails the targeted stimulation of specific acupuncture points to harmonize meridians and address ailments in a minimally invasive and relatively painless manner, rendering it readily acceptable to patients. Postural control for dysphagia post-stroke, derived from the Bobath technique, provides a neurodevelopmental framework for assessing and managing positional, motor, and functional deficits stemming from central nervous system injury (9). This approach prioritizes rectifying aberrant postures, fostering the restoration and enhancement of normal postural alignment, and ultimately facilitating nerve function improvement.

This study aimed to assess the efficacy of posture control combined with electroacupuncture targeting specific acupoints including Xiaguan (ST7), Jiache (ST6), Chengjiang (CV24), Lianquan (CV23), Jinjin (EX-HN12), Fengchi (GB20), and Yifeng (TE17) for the treatment of dysphagia post-stroke (10). The objective was to provide a scientific and objective evaluation of the treatment outcomes and investigate the underlying mechanisms. This innovative and efficacious rehabilitation approach offers promise in improving the management of dysphagia following stroke, reducing the risk of complications such as pneumonia, and ultimately enhancing patient survival rates.

## Materials and methods

### Patients

This study enrolled a total of 138 patients admitted to The Third Clinical Medical College of the Three Gorges University, Gezhouba Central Hospital of Sinopharm. These patients met both the diagnostic and inclusion criteria and were randomly assigned to either the observation group (*n* = 69) or the control group (*n* = 69). The group assignments followed the principles of randomized controlled trial (RCT) design. The study participants were individuals who experienced dysphagia during the recovery period after stroke. A few participants dropped out of the randomized clinical trial for various reasons. 5 participants in the observation group withdrew due to cerebral hemorrhage, while in the control group, 4 participants were lost to follow-up due to exacerbated pulmonary infection, and 2 participants were excluded from the study due to gastrointestinal bleeding during treatment. Ultimately, the observation group comprised 65 patients, and the control group comprised 62 participants.

### Inclusion and exclusion criteria

The inclusion criteria utilized for participant selection in this study were as follows: (1) patients meeting the diagnostic criteria for stroke, as defined by both Traditional Chinese Medicine (TCM) (aligned with the “Scoring Standards for Stroke Diagnosis” issued by the Encephalopathy Collaborative Group of the State Administration of Traditional Chinese Medicine) and Western medicine (diagnostic criteria for cerebral infarction or cerebral hemorrhage in the “Key Points for the Diagnosis of Various Major Cerebrovascular Diseases in China 2019”). Additionally, the duration of their illness should fall within the range of 15 days to 6 months; (2) patients diagnosed with swallowing dysfunction based on the water swallowing test; (3) swallowing dysfunction confirmed through swallowing contrast examination; (4) absence of any other neurological diseases; and (5) patients with stable vital signs who were willing to cooperate with therapists for rehabilitation training and examination.

Exclusion criteria were defined as follows: (1) patients with concomitant conditions such as oropharyngeal lesions (e.g., pharyngitis, pharyngeal tumors, posterior pharyngeal wall abscesses), esophageal lesions (e.g., esophagitis, esophageal cancer, cardiac spasm), or psychiatric disorders (e.g., functional neurological disorders); (2) patients with ulcers or a predisposition to bleeding easily; (3) patients with organ failure that could significantly impact their overall health; and (4) individuals experiencing concurrent consciousness and mental disorders.

Based on the preliminary results of the pre-experiment, the treatment group exhibited an effective rate of 90%, while the control group showed an effective rate of 70%. Assuming a probability of type I error (α) of 0.05 and a power (1-β) of 90%, an estimation of the required sample size for the study was conducted. The sample size calculation was performed based on the primary efficacy outcome measures, following the design of the clinical trial study, using the formula outlined below.


$$n=\frac{p_{1}\times \left(1-p_{1}\right)+p_{2}\times \left(1-p_{2}\right)}{{\left(p_{1}-p_{2}\right)}^{2}}\times {\left(\mu_{\alpha /2}+\mu_{\beta }\right)}^{2}$$

Where P1 = 90%, P2 = 70%, ɑ = 0.05, β = 0.1 are substituted into the formula.


$$n=\frac{0.9\times \left(1-0.9\right)+0.5\times \left(1-0.7\right)}{{\left(0.9-0.7\right)}^{2}}\times {\left(1.96+1.28\right)}^{2}$$

According to the above calculations, 63 cases are needed per group. Considering a 10% dropout rate, 69 cases should be enrolled per group. A total of 138 cases were reported in both groups.

### Eligible participants

**Figure 1:**
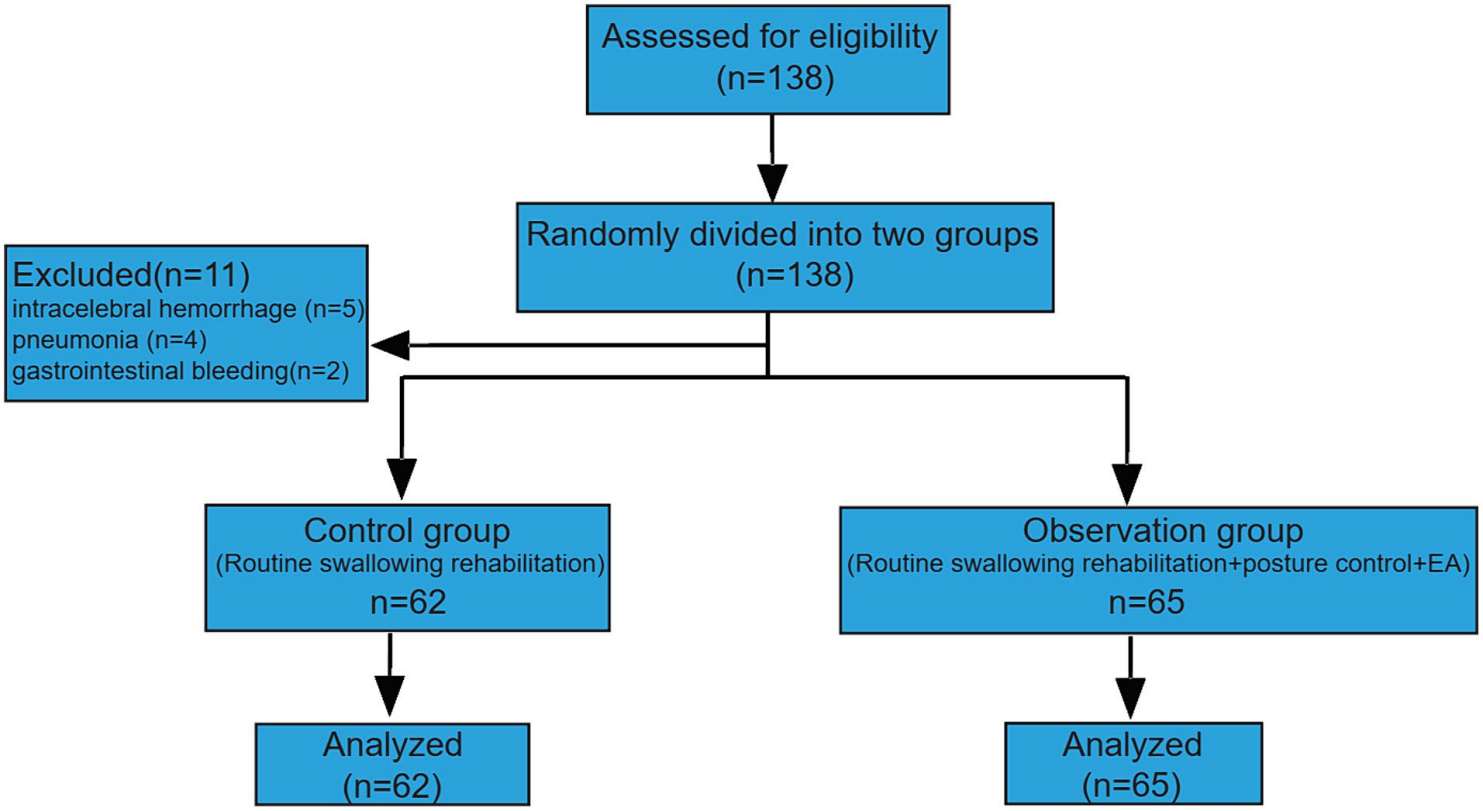
Schematic of the clinical trial.

A total of 138 patients with dysphagia after stroke were enrolled in this study, and 127 patients were included according to the inclusion and exclusion criteria (Figure 1).

### Interventions and assessment of outcomes

Conventional swallowing rehabilitation typically encompasses several components: (1) enhancement of the physical properties of food; (2) adjustment of eating position; (3) functional rehabilitation training for oropharyngeal and facial muscles, including sensory stimulation, oral and facial muscle strength training, coordination training for swallowing muscle movement, and swallowing skill training; (4) electrical stimulation of the pharyngeal muscles, including Neuromuscular Electrical stimulation (NMES), modified pharyngeal electrical stimulation (mPES) (7) and Pharyngeal electrical stimulation (PES) (6, 8, 11); (5) application of cold and hot stimulation to stimulate the pharyngeal mucosa; (6) Specific training for laryngeal elevation, hyoid muscle function, cricopharyngeal muscle function, pharyngeal cavity power, vocal cord function, and airway protection (11–13).

In terms of acupuncture point selection and electroacupuncture treatment, this study adhered to the principle of proximal acupoint extraction and targeted strategic points such as Xiaguan (ST7), Jiache (ST6), Chengjiang (CV24), Lianquan (CV23), Jinjin (EX-HN12), Fengchi (GB20), and Yifeng (TE17). Patients were positioned either seated or supine, and standard disinfection of the acupuncture points was performed prior to using disposable acupuncture needles measuring 0.25 mm × 40 mm for electroacupuncture application using conventional acupuncture techniques. To ensure effective stimulation of the acupoints, Han’s Acupoint Nerve Stimulator (LH202, Huawei Co. Ltd., Beijing, China) was employed, delivering a continuous wave with a current intensity of 1 mA and a frequency of 5 Hz. Each acupuncture session lasted for 30 min, and patients received treatment twice daily.

Posture control training methods comprised the following: (1) improving trunk stability and head–neck position: patients were guided to adopt a seated or supine position, with therapists positioned behind them to facilitate proper head and neck posture. This involved slight flexion of the head and extension of the trunk. To stabilize the hyoid bone, therapists placed their left hand on the patient’s jaw while palpating superficial large muscles such as the sternocleidomastoid and trapezius muscles, and loosening and stretching small muscles in inner and deep layers. In cases of muscle shortening, therapists addressed it by resisting jaw movements in various directions (up, down, left, and right), once daily for 15 min. This preparation was followed by head and neck control training. (2) Head and neck control training: this step encompassed passive, assistive, active, and resistive movements to achieve head-down, head-up, left–right rotation, left–right lateral flexion, and head indentation. The training involved a set of 10 movements, with 3 to 5 sets performed once daily based on the patient’s adaptation. Throughout the training, patients were encouraged to avoid holding their breath and were instructed to progress at their own pace, exerting their best effort during head control exercises. (3) Shrug exercise: patients raised both shoulders as close to their ears as possible for 10 s before simultaneously lowering them to the lowest position. It was emphasized that the patient’s upper limbs should remain straight during the shrugging motion. Similar to the head and neck control training, the shrug exercise consisted of a set of 10 movements, with 3 to 5 sets conducted once daily, depending on the patient’s adaptability.

### Water swallow test

During the treatment procedure, patients were provided with warm water at a temperature ranging from 37 to 40°C for consumption while in a seated or reclined position. Various levels were established to evaluate the patient’s capacity to swallow the warm water and the incidence of coughing:

Level I: patients successfully ingested 30 mL of warm boiled water in a single attempt without issue.

Level II: patients consumed 30 mL of warm boiled water across multiple attempts without experiencing choking or coughing.

Level III: patients managed to ingest warm water in one try but encountered coughing.

Level IV: patients required more than two attempts to ingest warm water and experienced choking.

Level V: patients were unable to consume the full 30 mL of water and frequently experienced coughing.

### Standardized swallowing assessment

During the initial assessment, several critical indicators were observed: (1) the patient’s level of consciousness and responsiveness to external stimuli were assessed; (2) the patient’s ability to control their trunk and maintain head stability was evaluated; (3) the presence of abnormal breathing patterns in the patient was noted; (4) the adequacy of the patient’s lip closure was examined; (5) the symmetry of the patient’s soft palate was assessed; (6) the functional status of the larynx was evaluated; (7) the integrity of the gag reflex and the patient’s autonomic coughing ability were checked for normalcy.

If none of the above indicators exhibit abnormalities, the subsequent assessment is divided into two parts. In the initial stage, the patient remains seated upright and is instructed to sequentially swallow 5 mL of water, repeated three times. During this process, the swallowing function is evaluated for potential issues, such as: (1) leakage of water from the mouth; (2) ineffective movement of the larynx; (3) repetitive movements during swallowing; (4) coughing or stridor between the larynx; (5) changes in voice (e.g., weakening, hoarseness, or inability to articulate) following water consumption. Upon successful completion of the first stage, the second stage is conducted, during which the patient is required to ingest 60 mL of water at once while maintaining an upright position for further assessment of the swallowing function. This stage involves evaluating the following aspects: (1) the ability to swallow 60 mL of water and the duration taken to complete the swallowing; (2) presence of coughing during or after swallowing; (3) occurrence of laryngeal stridor during or after swallowing; (4) any changes in voice following water consumption (e.g., weakening, hoarseness, or inability to articulate); (5) presence of aspiration.

### Videofluoroscopic swallowing study

To prevent alveolar deposition post-aspiration and safeguard the patient’s respiratory function, it is imperative to select a water-soluble barium sulfate suspension that can be absorbed effectively. The preparation of the suspension involves mixing barium sulfate powder with an appropriate quantity of water. Typically, 200 mg of barium sulfate is combined with 286 mL of water to achieve a homogeneous solution with a concentration of approximately 60%. This suspension is subsequently blended with pastes (thickening material) to create barium with varying consistencies. Contrast foods encompass diluted liquids (resembling water), pastes (similar to yogurt), and solids (akin to pudding). For contrast imaging, the PHILIPS multifunctional digital gastrointestinal machine (Essenta Rc system) is utilized. Under this equipment, the patient sequentially swallows 2 mL, 5 mL, and 10 mL of liquid ranging from diluted to thick consistency. Subsequently, the swallowing process is meticulously observed and assessed for further evaluation.

The general scoring criteria for the degree of dysphagia are as follows:

Oral phase: (1) inability to propel food from the mouth into the throat, resulting in food spillage from the lips or requiring forceful measures to initiate swallowing, is assigned a score of 0. (2) incapacity to form cohesive food boluses for passage into the throat, leading to food dispersion in the throat, receives a score of 1. (3) if residue remains in the mouth after swallowing attempts, it is scored as 2. (4) successful transfer of food into the throat with a single swallow is assigned a score of 3. Pharyngeal phase: (1) inadequate elevation of the throat, epiglottic malfunction, incomplete closure of the soft palate arch, and insufficient swallowing reflex result in a score of 0. (2) presence of significant residual food in the pharyngeal cavity and piriform recess warrants a score of 1. (3) minimal residue storage and complete clearance achieved through repeated swallows are given a score of 2. (4) successful passage of food into the esophagus with a single swallow is assigned a score of 3.

Degree of aspiration: (1) significant aspiration without accompanying cough is scored as 0 points. (2) presence of substantial aspiration leading to choking is assigned 1 point. A small degree of aspiration without coughing receives 2 points. (3) occurrence of minor aspiration with choking is rated 3 points. (4) absence of any aspiration is scored as 4 points.

The Videofluoroscopic Swallow Study (VFSS) is assessed on a scale ranging from 0 to 10, where a score of 0 indicates severe dysphagia, 2–3 suggests moderate dysphagia, and 7–9 signifies mild dysphagia.

### Statistical analysis

We established the database using Excel software, and SPSS 25.0 software was utilized for statistical analysis. Counting data were expressed as a percentage, and the measurement data was expressed as (x̅±s). Students *T*-test, χ^2^ test, Fisher’s exact test, Two-way ANOVA followed Turkey’s multiple comparisons test were conducted to analyze the difference. *p* < 0.05 indicates that the difference is statistically significant.

## Results

### The demographic and clinical characteristics of the treatment group were compared with those of the control group

Table 1 presents the clinical characteristics of the participants, encompassing gender, age, mean body mass index (BMI), educational status, presence of diabetes, and stroke type. The analysis indicated no statistically significant differences both between and within groups (Table 1).

**Table 1:** Demographic and clinical characteristics of the study participants.

Items	Study group		*p*
	Control (*n* = 62)	Observation (*n* = 65)	
Gender			
Male	35 (56.45%)	40 (61.54%)	0.560
Female	27 (43.55%)	25 (38.46%)	
Age (years, mean ± SD)	65.01 ± 9.85	66.35 ± 9.84	0.640
Course (months, mean ± SD)	1.97 ± 1.60	1.73 ± 1.44	0.553
Education status			
Junior high school and below	31 (50.00%)	35 (53.85%)	0.159
Senior high school	22 (35.48%)	27 (41.54%)	
College and above	9 (14.52%)	3 (4.61%)	
Smoke			
Yes	29 (46.77%)	30 (46.15%)	0.944
No	33 (53.23%)	35 (53.85%)	
Diabetes mellitus			
Yes	14 (22.58%)	20 (30.77%)	0.298
No	48 (77.42%)	45 (69.23%)	
Stroke type			
Cerebral infarction	46 (74.19%)	51 (78.46%)	0.571
Cerebral hemorrhage	16 (25.81%)	14 (21.54%)	

Values were expressed as N (percentage [%]). x̅ ± s mean plus or minus the standard deviation. *p* values were derived from the χ^2^ test, Students *T*-test or Fisher’s exact test.

### The combination of postural control and electroacupuncture significantly enhances recovery in patients suffering from dysphagia following stroke

**Figure 2:**
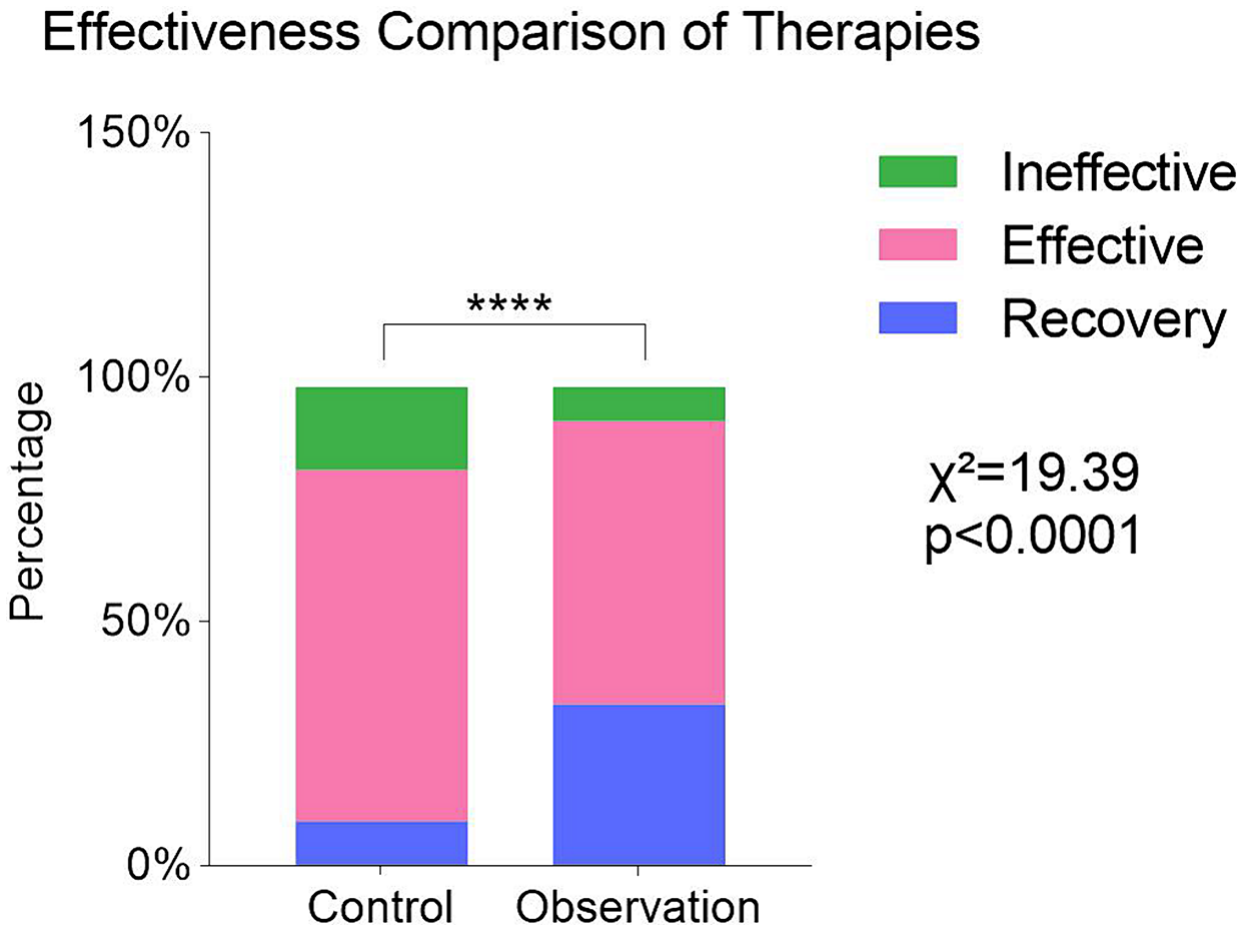
Comparison of clinical effectiveness in the treatment of dysphagia between the two groups. Values were expressed as N (percentage [%]). *p* value was derived from the χ^2^ test. (χ^2^,df) = (19.39,2),****p < 0.0001.

Compared to the control group, the observation group displayed a notably higher total treatment response rate (*p* < 0.0001). The total effective rate was 82.26% in the control group and 92.31% in the observation group (Figure 2). These findings indicate that the observation group, receiving a combination of conventional swallowing rehabilitation training, posture control, and electroacupuncture, exhibited superior treatment efficacy compared to the control group, which received conventional swallowing rehabilitation training only.

**Figure 3:**
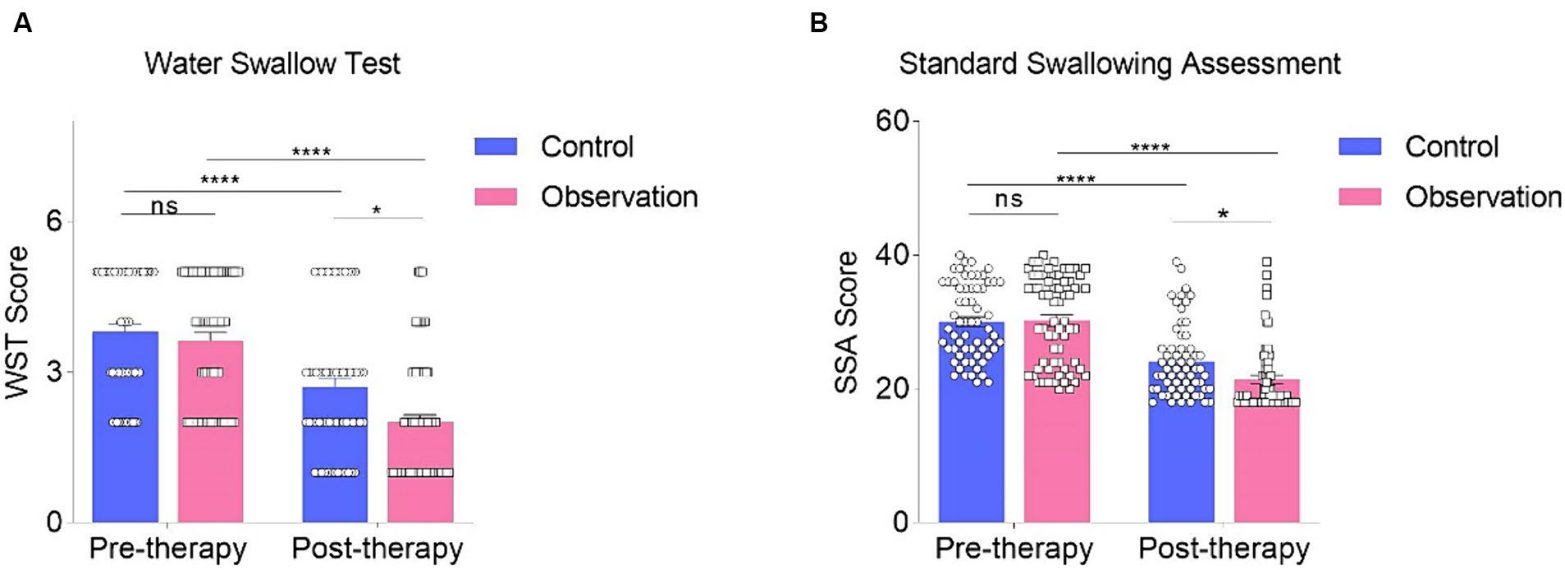
Comparison of pre- and post-treatment water swallow test **(A)** and standard swallowing assessment **(B)** was conducted using Two-way ANOVA followed by Tukey’s multiple comparisons test. * *p* < 0.05, **** *p* < 0.0001, and “ns” indicates no significance.

We then assessed and compared standardized swallowing assessment (SSA), water swallow test (WST), and aspiration tests between the two groups. Both groups demonstrated some degree of recovery after treatment, with a significant difference observed between the two groups (*p* < 0.05, Figure 3A).

The SSA scores in both the observation group and control groups decreased after treatment, and this change was statistically significant (*p* < 0.0001, Figure 3A). Before treatment, there was no significant difference between the observation and control groups (*p* > 0.05, Figure 3A). However, after the intervention, a significant difference was observed between the control group and the observation group (Figure 3A). The WST results were compared between the two groups by calculating the number of individuals at each stage of WST in both groups. The evaluation was conducted using Two-Way ANOVA. When comparing the results before and after treatment in the two groups, a significant difference was found (*p* < 0.0001, Figure 3B), indicating that both treatment methods had a therapeutic effect. Furthermore, no significant difference in WST results was found before treatment between the two groups (*p* > 0.05, Figure 3B). However, after treatment, the observation group exhibited remarkable improvements compared to the control group (*p* < 0.05, Figure 3B). The combination of conventional swallowing rehabilitation training, posture control, and electroacupuncture was more effective than conventional swallowing rehabilitation training alone in improving water swallow function (Figure 3B).

**Figure 4:**
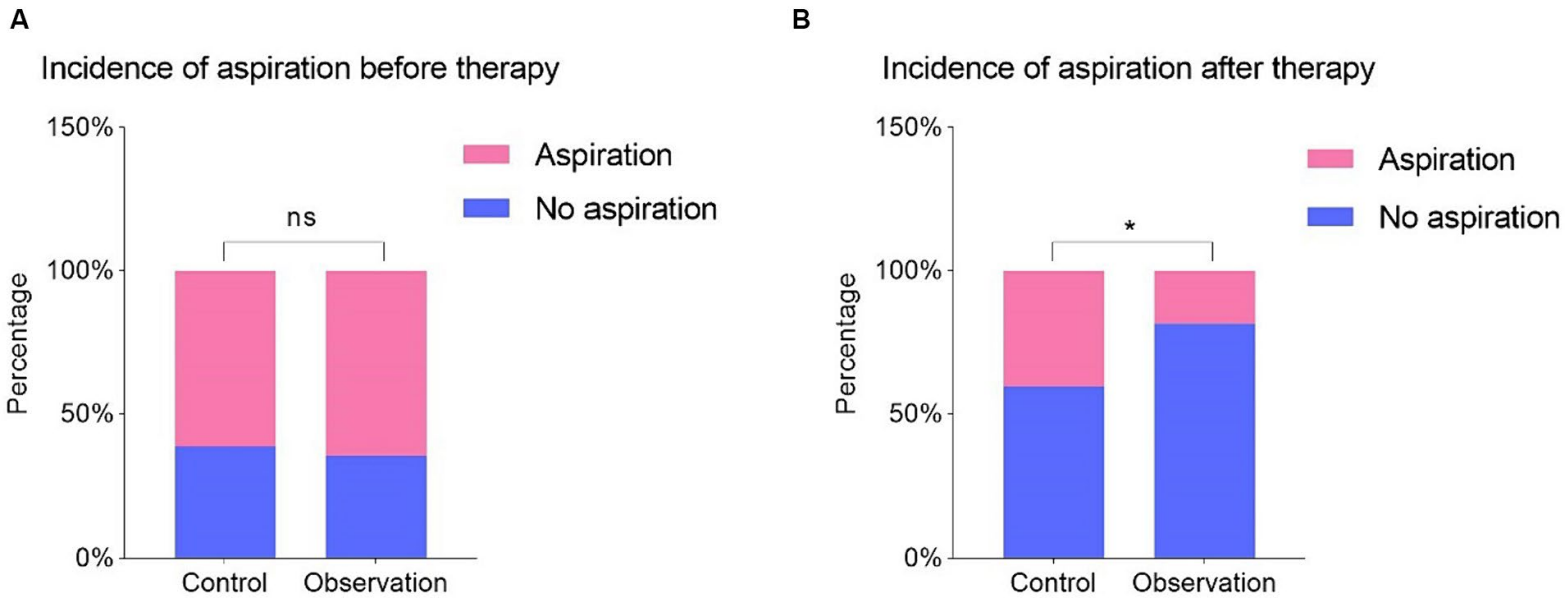
Comparison of whether there is aspiration before **(A)** and after **(B)** treatment in two groups. Values were expressed as N [percentage (%)]. *p* value was derived from the Fisher’s exact test. *p  <  0.05, and “ns” indicates no significance.

Regarding the aspiration test, there was no significant difference between the two groups before treatment (Figure 4A). However, after treatment, a significant difference emerged between the two groups (*p* < 0.05, Figure 4B), with the observation group demonstrating greater improvement in aspiration compared to the control group. These findings indicate that patients in the observation group experienced substantial enhancements in swallowing function after treatment compared to those in the control group.

**Figure 5:**
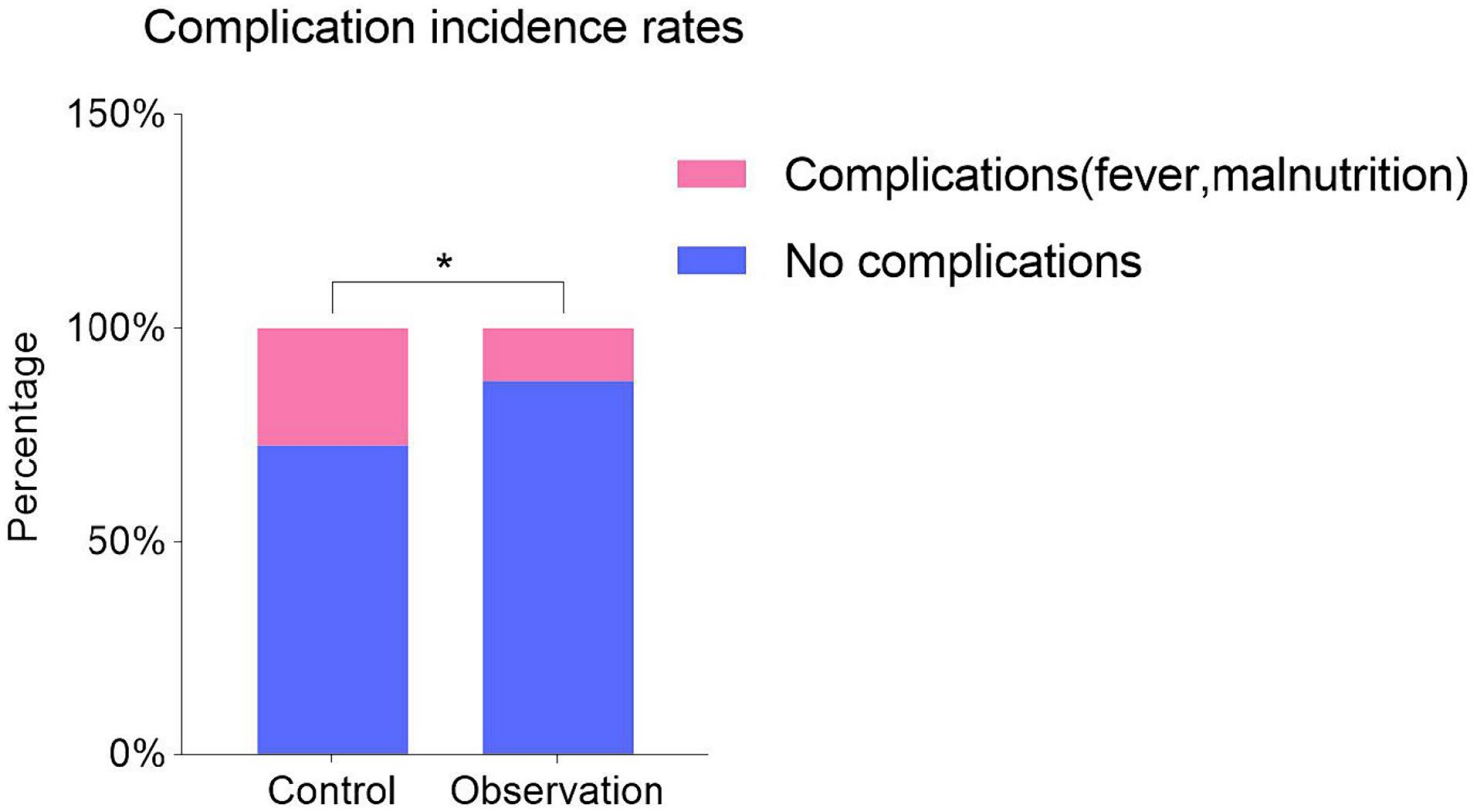
Comparison of complications (fever and malnutrition) in two groups after treatment. Values were expressed as N (percentage [%]). *p* value was derived from Fisher’s exact test. * *p* < 0.05.

To assess the safety of the treatment and the occurrence of complications post-treatment, we evaluated fever and nutritional disorders in both groups. In contrast, among the 62 cases in the control group, 11 participants experienced fever, and 7 exhibited nutritional disorders, leading to a total incidence of 29.03%. Among the 65 cases in the observation group, only 8 participants developed fever, while none showed nutritional disorders, resulting in a total incidence of 12.13%. Statistical analysis revealed a significant difference between the two groups (*p* < 0.05, Figure 5). These findings suggest that the observation group had a lower incidence of fever and nutritional disorders compared to the control group, indicating the safety and benefits of the intervention in the observation group.

## Discussion

Stroke is a medical condition resulting from disruptions in cerebral blood circulation, leading to ischemia and hypoxic lesion necrosis of brain tissue (14). The main pathogenesis is associated with infarction of cerebral blood vessels or sudden cerebral vascular hemorrhage, manifesting clinically as neurological loss corresponding to the affected lesion (14–17). However, when dysphagia occurs after a stroke, cerebrovascular ischemic injury damages the nerve and central functions related to swallowing (18), resulting in slow tongue movement and Swallowing muscle coordination dysfunction (19). Patients with dysphagia after stroke also suffer from additional complications, such as Speech impediment, malnutrition, Electrolyte imbalance, and aspiration (20). In particularly, aspiration can lead to lung infections, significantly impacting the prognosis of stroke patients (9, 18, 20). Therefore, effectively treating these complications after stroke is of great significance to promote physical and neurological recovery (21). However, conventional rehabilitation training and medication guidance have not shown significant efficacy in improving swallowing dysfunction (22).

Electroacupuncture, rooted in traditional Chinese medicine theory, involves stimulating meridian acupuncture points. This technique offers strong penetration and regulation of body functions (23). It enhances the immune system, balances Yin and Yang, and promotes longevity (24, 25). Previous studies indicate that acupuncture points for treating swallowing disorders after stroke include Lianquan (CV23), Tiantu (CV22), Chengjing (BL56), Chejia (ST6), Yifeng (TE17), Taixi (KI3), Fengchi (GB20), Neiguan (PC6), Sanyinjiao (SP6), Yintang (EX-HN3), Taiyang (EX-HN5), Jinjin (EX-HN12), Yuye (EX-HN13), Zusanli (ST36), Fenglong (ST40), Taichong (LR3), Xiaguan (ST7) (26). Among these, the most commonly used acupuncture points are Lianquan (CV23), Fengchi (GB20), Yifeng (TE17), Jinjin (EX-HN12), and Yuye (EX-HN13) (27).

In this study, adhering to the principle of traditional Chinese medicine theory and Meridian theory (28), the treatment for swallowing disorders after stroke primarily targets acupuncture points on the head and neck, such as Xiaguan (ST7) acupoint, and Jiache (ST6) acupoints, and Chengjiang (CV24) acupoint on the face (29), Lianquan (CV23) acupoint, and Jinjin (EX-HN12) acupoints, and Yuye (EX-HN13) acupoint on the tongue pharynx (30), as well as the Fengchi (GB20) and Yifeng (TE17) acupoints on the neck (31). The Chengjiang (CV24) acupoint’s proximity to the mouth makes it effective in treating mouth and adjacent area disorders, which is associated with the Yin and Yang theory (32). Electroacupuncture at Lianquan (CV23) acupoints promotes the contraction of swallowing muscles and local blood circulation (33). Additionally, stimulation of the Fengchi (GB20) and the Yifeng (TE17) acupoints improves the blood rheology, peripheral microcirculation, blood flow of the brain, and skull base blood flow (33, 34), and establishes collateral circulation at the lesion to improve the blood sample supply of brain tissue as soon as possible, thereby accelerating the recovery of central nervous system function and rebuilding upper motor neurons, so that the motor nucleus of the medulla oblongata can be reasonably innervated (35). Besides, Lianquan (CV23) acupoint as a peripheral stimulation strategy, could improve the swallowing function in PSD model mice through the activation of motor cortex inputs to the NTS through the PBN. the application of EA-CV23 for the treatment of PSD by addressing how motor cortex activation benefits recovery of swallowing function via a subcortical pathway, suggesting that acupuncture treatment could be used as an effective therapeutic intervention to improve swallowing function (27).

Postural control for dysphagia after stroke is derived from the Bobath technique, which employs a neurodevelopmental approach (9). Its primary objective is to enhance nerve function by inhibiting abnormal posture by inhibiting abnormal postures, thereby promoting the development and recovery of normal posture (36). This approach proves effective in addressing stroke-related swallowing disorders as it enhances the patient’s ability to control ability of body position through targeted training of the upper limbs, trunk, head, and neck (37). By holistically training multiple body parts, it creates a safe environment for patients experiencing dysphagia to eat (38), improves muscle tone, maintains the normal anatomical position of the articulation organs, and synergizes with comprehensive rehabilitation treatment to more efficiently alleviate swallowing disorders (39). The findings of this study reveal that combining posture control with electroacupuncture and conventional rehabilitation training yields notable improvements in post-stroke dysphagia, achieving an overall treatment rate of 92.31%, significantly surpassing the control group. Moreover, subsequent evaluations of indicators, including SSA, WST, and aspiration, demonstrate improved swallowing function in both groups, with the observation group displaying more significant results than the control group. Additionally, based on the evaluation of fever and nutritional disorders, the observation group exhibited superior effects.

### Limitations of the study

The sample size is small and comes from a single source, consisting solely of patients from The Third Clinical Medical College of the Three Gorges University, Gezhouba Central Hospital of Sinopharm. Hence, larger-scale, multi-regional studies are warranted to validate our findings. Furthermore, no research on the efficacy of routine rehabilitation, posture control and electroacupuncture in treating dysphagia after stroke has been carried out. Therefore, the results should be interpreted with caution. Future studies should expand the sample size and groups to provide a more comprehensive understanding of the effectiveness of electroacupuncture and postural control.

## Conclusion

In summary, both approaches—routine rehabilitation training and posture control combined with electroacupuncture based on routine rehabilitation training—show promising results in treating patients with swallowing disorders after stroke. These interventions enhance swallowing function and reduce the occurrence of complications such as aspiration, fever, and nutritional disorders. Notably, the combination of electroacupuncture, posture control, and routine rehabilitation training proves to be more effective than routine rehabilitation training and provides a more favorable treatment outcome.

## Data availability statement

The original contributions presented in the study are included in the article/supplementary material, further inquiries can be directed to the corresponding author.

## Ethics statement

The studies involving humans were approved by Gezhouba central hospital of Sinopharm Ethics committee. The studies were conducted in accordance with the local legislation and institutional requirements. The participants provided their written informed consent to participate in this study.

## Author contributions

YW: Writing – original draft, Validation, Supervision. ZZ: Writing – original draft, Data curation. QL: Writing – original draft, Software, Methodology. XY: Writing – original draft, Data curation. JR: Writing – original draft, Formal analysis. YC: Writing – original draft, Project administration, Investigation. HZ: Writing – review & editing, Writing – original draft, Resources, Funding acquisition, Conceptualization.
